# Supplementary material for: The interactome of intact mitochondria by cross-linking mass spectrometry provides evidence for coexisting respiratory supercomplexes
Source: Mol Cell Proteomics. 2017 Dec 8;17(2):216–32. doi: 10.1074/mcp.RA117.000470 (PMC5795388; doi:10.1074/mcp.RA117.000470)
Supplement: Supplemental Data [file supp_RA117.000470_133922_0_supp_23978_fzffwf.zip › spectra_annotation/mito_spectra_annotation/75-1-5-1-11-1.pdf]

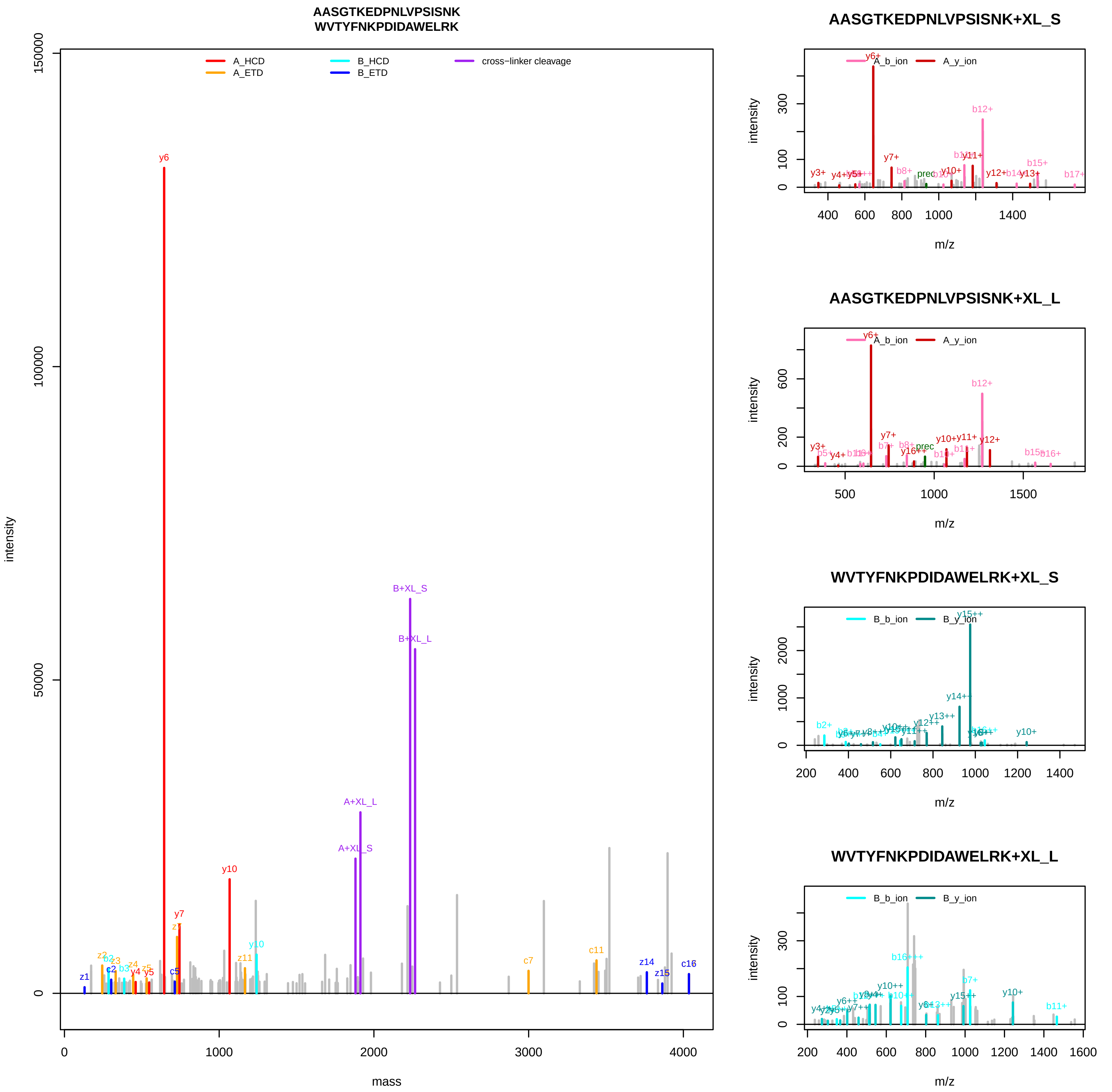
AASGTKEDPNLVPSISNK
WVTYFNKPDIDAWELRK
A_HCD
A_ETD
B_HCD
B_ETD
cross−linker cleavage
intensity
mass
150000
100000
50000
0
1000
2000
3000
4000
y6
y10
y7
z7
B+XL_S
B+XL_L
A+XL_L
A+XL_S
y10
z11
c7
c11
z14
z15
c16
c5
z1
c2
b2
z2
z3
b3
z4
y4
z5
y5
AASGTKEDPNLVPSISNK+XL_S
AASGTKEDPNLVPSISNK+XL_L
WVTYFNKPDIDAWELRK+XL_S
WVTYFNKPDIDAWELRK+XL_L
A_b_ion
A_y_ion
B_b_ion
B_y_ion
intensity
m/z
y6+
b12+
y7+
y3+
y10+
y11+
b15+
b17+
y12+
y13+
prec
b16+
y15++
y14++
y13++
y12++
y10+
b2+
b16+++
b7+
b11+
y15++
